# Predicting the Effects of Repetitive Transcranial Magnetic Stimulation on Cognitive Functions in Patients With Alzheimer's Disease by Automated EEG Analysis

**DOI:** 10.3389/fncel.2022.845832

**Published:** 2022-05-19

**Authors:** Cihan Bilge Kayasandik, Halil Aziz Velioglu, Lutfu Hanoglu

**Affiliations:** ^1^Department of Computer Engineering, School of Engineering and Natural Sciences, Istanbul Medipol University, Istanbul, Turkey; ^2^Department of Women's and Childrens' Health, Karolinska Institutet, Stockholm, Sweden; ^3^Functional Imaging and Cognitive-Affective Neuroscience Lab (fINCAN), Regenerative and Restorative Medicine Research Center (REMER), Health Sciences and Technology Research Institute (SABITA), Istanbul Medipol University, Istanbul, Turkey; ^4^Department of Neurology, School of Medicine, Istanbul Medipol University, Istanbul, Turkey

**Keywords:** Alzheimer's disease, machine learning, support vector machine, repetitive transcranial magnetic stimulation (rTMS), personalized treatment, artificial neural network

## Abstract

Alzheimer's disease (AD) is a progressive, neurodegenerative brain disorder that generally affects the elderly. Today, after the limited benefit of the pharmacological treatment strategies, numerous noninvasive brain stimulation techniques have been developed. Transcranial magnetic stimulation (TMS), based on electromagnetic stimulation, is one of the most widely used methods. The main problem in the use of TMS is the existence of large individual variability in the results. This causes a waste of money, time, and more importantly, a burden for delicate patients. Hence, it is a necessity to form an efficient and personalized TMS application protocol. In this paper, we performed a machine-learning analysis to see whether it is possible to predict the responses of patients with AD to TMS by analyzing their electroencephalography (EEG) signals. For that purpose, we analyzed both the EEG signals collected before and after the TMS application (EEG1 and EEG2, respectively). Through correlating EEG1 and repetitive transcranial magnetic stimulation (rTMS) outcomes, we tried to see whether it is possible to predict patients' responses before the treatment application. On the other hand, by EEG2 analysis, we investigated TMS impacts on EEG, more importantly if this impact is correlated with patients' response to the treatment. We used the support vector machine (SVM) classifier due to its multiple advantages for the current task with feature selection processes by stepwise linear discriminant analysis (SWLDA) and SVM. However, to justify our numerical analysis framework, we examined and compared the performances of different feature selection and classification techniques. Since we have a limited sample number, we used the leave-one-out method for the validation with the Monte Carlo technique to eliminate bias by a small sample size. In the conclusion, we observed that the correlation between rTMS outcomes and EEG2 is stronger than EEG1, since we observed, respectively, 93 and 79% of accuracies during our data analysis. Besides the informative features of EEG2 are focused on theta band, it indicates that TMS is characterizing the theta band signals in patients with AD in direct relation to patients' response to rTMS. This shows that it is more possible to determine patients' benefit from the TMS at the early stages of the treatment, which would increase the efficiency of rTMS applications in patients with Alzheimer's disease.

## Introduction

Alzheimer's disease (AD) is the most common type of dementia, characterized by a progressive loss of cognitive ability (Lane et al., 2018). The most prevalent signs of Alzheimer's disease include memory and orientation problems, as well as executive and motor dysfunctions (Guarino et al., 2019). At the molecular level, AD has been linked to synaptic impairment, as well as amyloid-beta and tau protein buildup (Bloom, 2014). As a result of this disease's progressive nature, loss of cognitive and motor function eventually leads to death.

Because there is currently no cure for AD, healthcare providers must rely on treatments aimed at delaying the illness's progression and lowering cognitive damage. The majority of these therapeutic methods rely on pharmacological substances (Cummings et al., 2019). Pharmacological medicines, on the other hand, are unable to provide an effective treatment for Alzheimer's disease, and, as a result of their extensive side-effect profile, they also cause other issues (Sharma, 2019). As a result, scientists have begun to investigate options other than pharmaceutical medications for treating this disease.

Transcranial magnetic stimulation (TMS), which has long been used successfully in the treatment of depression (Sonmez et al., 2019), is now being employed as a possible alternative treatment for other neurodegenerative diseases such as Alzheimer's disease (Freitas et al., 2011; Mimura et al., 2021), Parkinson's disease, and other dementias (Begemann et al., 2020; Hanoglu et al., 2021). TMS is a noninvasive approach that uses a magnetic field created outside the body to stimulate or inhibit specific areas of the brain by applying it to the scalp (Hamid et al., 2019). In some ways, TMS treatment appears to be superior than the pharmaceutical approach. TMS can be favored over pharmaceutical medicines due to its ease of use, low side effect profile, and excellent behavioral and cognitive benefits.

However, the effect of TMS on brain activity has not been fully understood (Sale et al., 2015). It is observed that some patients respond positively to treatment, while other gives no response (Dunlop et al., 2016). Unfortunately, this heterogeneity of treatment responses among patients has no obvious connection with the clinical measurements such as personality, demographic, sex, and age (Bailey et al., 2019). Hence, advanced computational methods are needed to analyze the connection between patients' certain properties and the TMS treatment to predict the patients' possible response to the treatment. That is necessary to generate efficient treatment strategies.

There are some studies with the purpose of predicting the TMS response of patients with psychiatric diseases. Chekroud et al. attempted to match depression patients with the treatment according to the patients' Quick Inventory of Depressive Symptomatology (QIDS) test rates (Chekroud et al., 2016). Another study analyzed structural magnetic resonance imaging results to predict the treatment outcomes of patients with schizophrenia (Koutsouleris et al., 2018). There are other studies focus on predicting the outcomes of treatments other than TMS, which are out of our focus on this study (Garnaat et al., 2019).

On the other hand, there are only a few studies on machine-learning analysis of EEG in predicting the effectiveness of rTMS. Corlier et al. (2019) generated a predictive model based on the functional connectivity on EEG, and as our purpose, they tried to obtain the most informative features for the rTMS benefit prediction. For the classification task, they used Elastiknet (Zou and Hastie, 2005), which randomly selects the subset of features and tries to maximize the classification accuracy over all random trials. Hasanzadeh et al. combined linear and non-linear features from EEG to distinguish TMS responders from non-responders, again on patients with depressive disorders. They also used the multi-source nature of the EEG data and tried to see the most significant electrode locations beside most significant EEG features. They used kNN classifier which is a primitive and low-capacity method that would fail on slightly complicated tasks (Hasanzadeh et al., 2019). Bailey et al. analyzed the electroencephalogram (EEG) signal of patients with depression to see whether it is possible to predict patients' response to rTMS treatment. They used band power and connectivity features with a linear SVM classifier to generate their prediction model (Bailey et al., 2019).

Besides these studies focusing on depressive disorders, to the best of knowledge, there is no significant attempt to generate a computational model for EEG analysis with the purpose of predicting the TMS response of patients with AD. Since AD is a neurodegenerative disease, the TMS therapy for patients with AD have more factors to be considered than depressive disorders. Besides, TMS effect is more variable in patients with AD than patients with depressive disorders (Iimori et al., 2019; Weiler et al., 2020). Hence, our task is more complicated, and the discussed methods above would not be applicable to the current task.

The EEG forms a critically high-dimensional dataset whereas it is highly challenging to collect a large number of samples for neurological disorders. Hence, the EEG data analysis for cognitive neuroscience studies is highly challenging. Due to the limited number of samples, studies in the literature mostly reduce the EEG data to a very low dimension by ignoring the advantage of the multi-source nature of EEG data.

**Figure 1:**
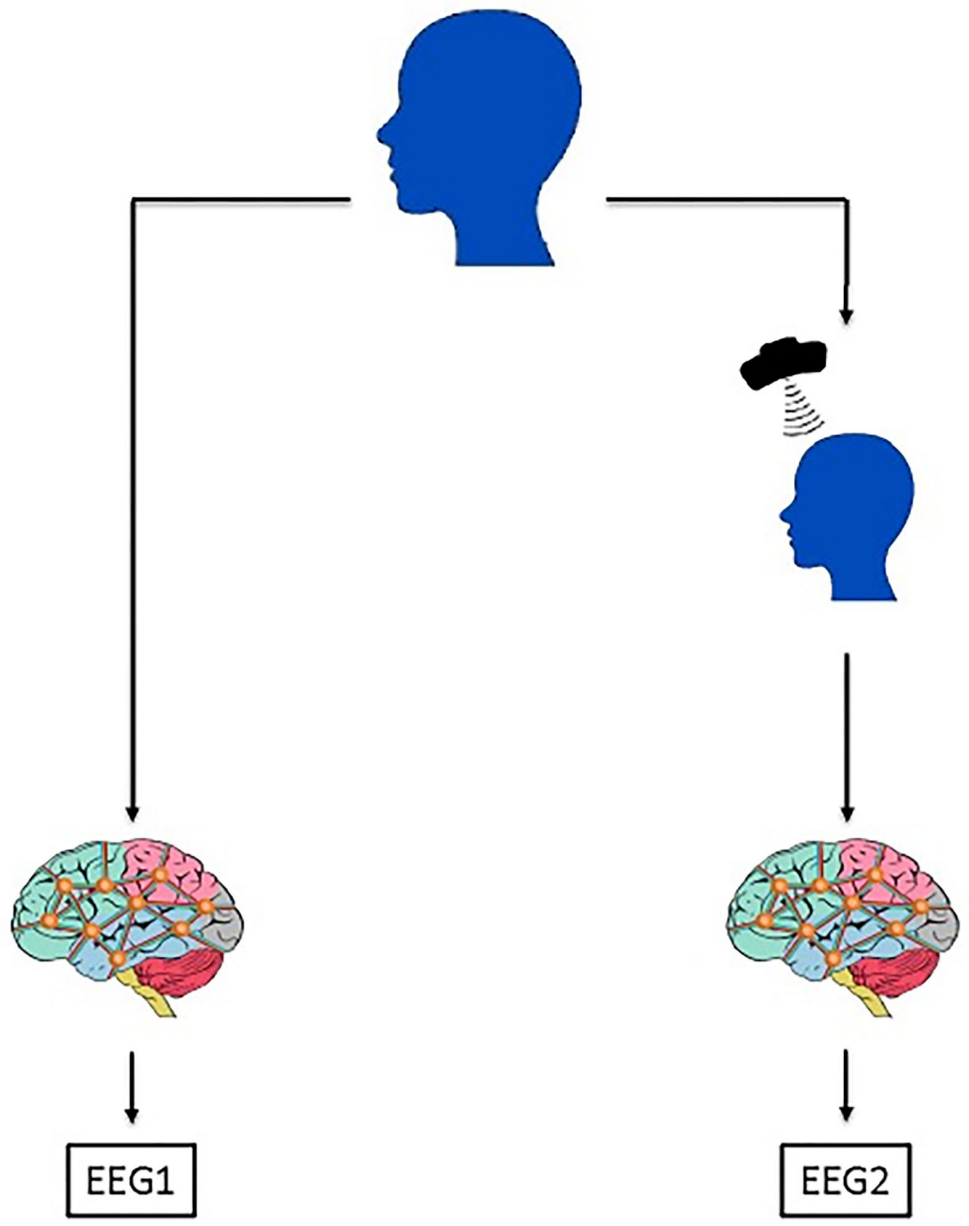
The framework of the data acquisition process. For each patient, EEG signal collection is applied once before the TMS treatment application, that EEG signal is labeled as EEG1 in the manuscript. Another EEG signal is collected after the TMS treatment application which is labeled as EEG2. In this study, EEG1 and EEG2 were separately analyzed. Through the EEG1, we gather the information if it is possible to predict the patients' response to the treatment. On the other hand, by the analysis of EEG2, we sought for the effects of TMS application on patients' EEG signal if it is dependent on the patients' benefit from the treatment.

In this study, we examined the possibilities to maximize the efficiency of the TMS treatments by predicting patients' responses before the TMS application by focusing on preserving the advantage of the multi-source nature of the EEG data. For that purpose, we analyzed pre-TMS EEG signals, which will be named as EEG1 throughout the paper for simplicity. Besides that, we investigated whether a dose application of TMS would increase the correlation between EEG2 and rTMS. For that purpose, we analyzed the post-TMS EEG signals, which will be named as EEG2 in the paper for simplicity (Figure 1). Throughout our analysis besides generating a prediction model, we seek more insight on EEG and TMS relation in patients with AD. For that purpose, we analyzed EEG1 and EEG2 from various perspectives. We measured the sensitivity and stability of the correlations between EEG1/EE2 and rTMS outcomes with respect to the small sample number. We applied multiple dimension reduction and classification methods, compared their performances, and interpreted the results.

The rest of the paper is organized as follows: section Materials and Methods provides the details of the dataset collection, data preprocessing, and the brief explanation of applied feature selection and classification techniques. It explains the process of generating the ground-truth labels and optimizing the machine-learning algorithms. Section Results describes all the applied data analysis steps. It explains our motive for further data analysis for more insight on the effects of TMS treatment on patients' EEG signals, the sensitivity measure of the data analysis process, comparative results of feature selection, and classification algorithms. Section Discussion discusses the obtained machine-learning results. It explains the interpretation of these results from a clinical view and lists the future motivation for this study.

## Materials and Methods

### Subjects

To determine the minimum sample size of patients with AD to be applied TMS, G^*^power (version 3.1.6.6) software was used. We discovered that for 90% power with a significance level of = 0.05, a sample size of at least *n* = 13 participants is required, and thus, we decided to include at least *n* = 14 subjects in the investigation. Our research included 14 patients with AD (10 women, ages 56–84). Based on their responses to a TMS safety-screening questionnaire, all patients were eligible for TMS operations. The Ethical Committee of Istanbul Medipol University accepted our research with the number of Ethical Report: 10840098–604.01.01–E.45425. In Istanbul Medipol University Hospital, all patients and their relatives submitted written informed consent, and all processes were followed. During the study period, patients were assessed while on usual therapy with no changes to their medical treatment.

The clinical diagnosis of AD was made by an experienced neurologist according to the NINCDS-ADRDA (McKhann et al., 2011) criteria. The Clinical Dementia Rating Scale (CDR) scores of the patients participating in the study were between 1-2. The patients were using acetylcholinesterase inhibitors and/or memantine as a medicine. Exclusion criteria were defined as having metallic implants, unable to walk independently, being physically disabled, history of alcohol or substance abuse, mental illness including schizophrenia and delirium, and epileptic disease, seizure, brain tumor, or trauma.

### Labeling the Patients as TMS+ and TMS–

In the direction of deciding whether a patient is positively or negatively affected by the TMS treatment, we considered only the Mini-Mental State Examination (MMSE) scores of neuropsychometric tests (NPTs). For that purpose, if a certain patient's MMSE score increased after the TMS treatment, it was labeled as TMS+; similarly, if a certain patient's MMSE score decreased after the TMS treatment, it was labeled as TMS–. In our dataset, there were two patients with constant MMSE scores before and after the TMS treatment. There was no certain procedure for labeling these patients. However, during the classification analysis, we observed that their EEG signals behave like a TMS+ patient (by labeling them as TMS+ we increased the classification results by 20%). Hence, if the MMSE score was constant, we considered these patients as they benefit the treatment, hence TMS+.

### Experimental Design

Each patient underwent 10 sessions of rTMS therapy over the course of 2 weeks. Patients provided the following information 1 week prior to TMS. The Turkish version of the Mini-Mental State Examination (MMSE) scores was used to assess the overall cognitive status. We also acquired a resting-state EEG1 and EEG2 recording to assess which patient would benefit from the TMS application using machine learning. The MMSE and EEG data were taken from patients again after 2 weeks of TMS therapy.

### EEG Recording and Preprocessing

Electroencephalography recordings were applied in a dimly illuminated and electrically shielded room (Faraday cage). According to the international 10–20 system, EEG was enrolled with the Brain Amp amplifier DC system. EEG band level was between 0.01 and 250 Hz and sampling rate was 500 Hz. Recording electrodes were used with 32 Ag–AgCl electrodes. All electrodes were mounted by means of Easy-cap except reference, ground, and EOG electrodes. According to the head size of patients, we changed Easy-cap and chose a suitable one. One reference electrode was bonded with a latch to the left earlobe, and the other reference electrode was also bonded to the front of the right earlobe. The ground electrode was also stuck together behind the right earlobe. EOG electrodes were bonded to the right side of the forehead and to below the left eye. All electrode impedances were decreased to <15 kΩ (ground = 1 Ω).

Before and after TMS therapy, two spontaneous EEG data were collected from patients. The spontaneous EEG protocol was 4-min eyes-open recording and 4-min eyes-closed recording. A video recorder recorded all the EEG processes.

To prepare the EEG data for further analysis, we used Brain Vision Analyser software. Preprocessing steps included six different stages. First of all, using infinite impulse response (IIR) section, we filtered raw data low (0.1 Hz) and high cutoff (60 Hz) and enabled the notch filter (50 Hz). To remove the blink-reflex, we used independent component analysis (ICA) after then we performed inverse ICA command. We segmented the data manually as open eyes–closed eyes. Open and closed eyes data were recorded for 4 min. Using again segmentation, we created 1-s size of segments and skipped bad intervals. Finally, we rejected artifacts such as blink, and muscle movement manually and saved data in MAT format.

Manual artifact rejection was performed by examining each 1-s epoch one by one. Only one experienced person (HAV) performed the preprocessing processes to ensure standardization.

### Identification of Stimulation Locations and rTMS Parameters

We determined a stimulation coordinate for each patient using a 10–20 EEG system. We aim to stimulate P3 in other words the left posterior parietal cortex (Herwig et al., 2003).

For the TMS application, we used the MAG & More company's PowerMag Research tool. The coil type of the TMS device was an “eight” coil with a winding diameter of 2 x 70 mm. We assessed the motor threshold by stimulating the TMS primary motor region, which needed at least 5 of 10 consecutive pulses to cause contraction in the abductor pollicis brevis muscle, at the start of the first study week. TMS coils were held over the left parietal cortex with the handle-oriented 45 degrees to the midsagittal line, generating a posterior to anterior current (Brown et al., 2014). The coil was manually moved to the coordinates displayed on the neuro-navigation device. During the 20-min stimulation period, rTMS was delivered at a 100% resting-motor threshold (Hermiller et al., 2019). The rTMS procedure was 20 Hz for 2 s of stimulation duration (totaling 1,640 pulses each session), followed by a 28-sinterstimulus period (Wang et al., 2014).

### Data Analysis

**Figure 2:**
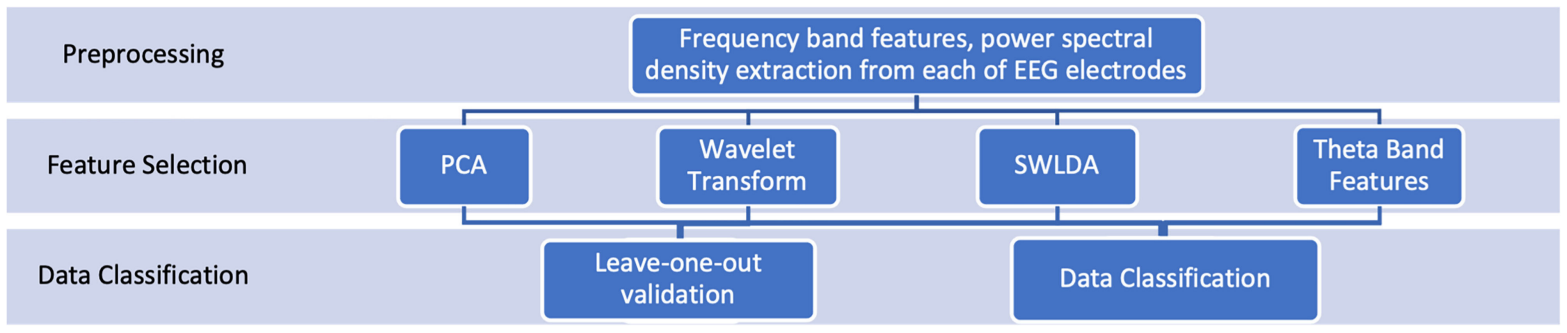
Analysis framework: Our analysis is based on frequency band features extracted from EEG1 and EEG2. To justify our numerical analysis framework, we examined and compared the performances of different feature selection and classification techniques. We reported all numerical results.

To justify our numerical analysis, we performed multiple feature selection and classification methods and compare their results. The complete analysis framework can be seen in Figure 2.

#### Feature Extraction

**Figure 3:**
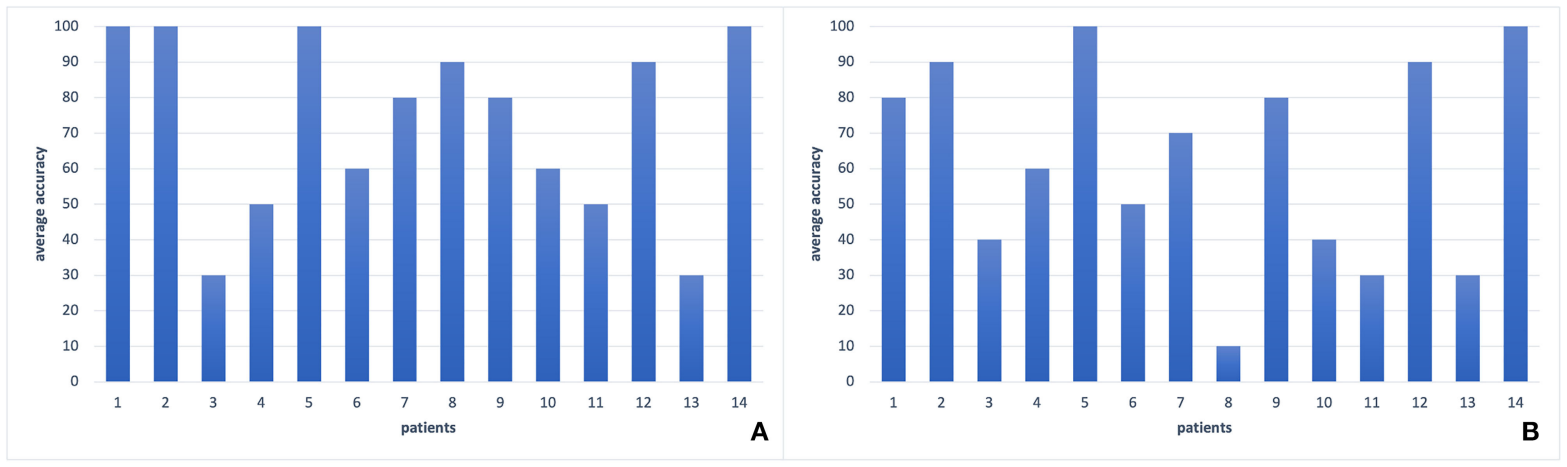
Comparison of performances of leave-one-out validation for different window sizes for feature extraction process. **(A)** Results with window size length 1 s, **(B)** Results with window size length 4 s. The results with window size length 2 s can be seen in Figure 5A. As it is seen in the Figure, 2-swindows give the best classification performance.

The raw form of the EEG signal is highly complicated and difficult to interpret. If we focus only on the analysis task, EEG is very large and possibly stores redundant information. Hence, a dimension reduction or feature extraction step is required before classification. In our study, first, we applied the fast Fourier transform to extract the wavelengths. In the literature, the EEG analysis focuses on five main frequency groups such as delta (0.5–4), theta (4–7), alpha (7.5–13), beta (15–28), gamma (29–48), and we followed the same procedure. For each of these wavelengths, we calculated the power spectral density as the magnitude squared of the filtered data. Then, we got the average power spectral density for non-overlapping windows with a length of 2 s. The feature extraction window size was numerically determined. We observed that the classification accuracy makes a peak when the feature extraction window size is set to 2 min (Table 1; Figure 3). Instead of obtaining the power of the frequency band, which is only a scaler, we used this new signal to represent the EEG data. By that, we formed a denser and more informative representation of EEG while preserving more information about the dataset.

**Table 1:** The effect of different feature extraction window sizes on the data classification performance.

	**Feature extraction with window size 1 s**	**Feature extraction with window size 2 s**	**Feature extraction with window size 4 s**
Average accuracy	73%	**78**%	59%

*The accuracies are recorded during the leave one out validation of EEG2. The maximum accuracy is in bold*.

After this basis feature extraction step, we examined the performance of different dimension reduction techniques such as wavelet transform, principal component analysis, and weight hierarchy determined by stepwise linear discriminant analysis and SVM classifier. Through these applications, we aimed to validate our numerical analysis and investigate the data from the different perspectives to get the core information from EEG signal. The contribution of these dimension reduction techniques to the performance of the data analysis is given in the Results section.

##### Principal Component Analysis

Principal component analysis (PCA) is one of the most widely applied dimension reduction techniques. The main idea of the PCA is finding the most informative dimensions of the data and projecting the data into the subspace by the determined informative dimensions (Jolliffe, 2014). For that purpose, mathematically, all eigenvalues of the D^T^D are determined, where D^T^D is called the covariance matrix of the dataset D. Covariance matrix basically shows how each feature is related to all others. Therefore, by the eigenvalues, PCA examines the features of the dataset and tries to find the most informative and independent ones. For that purpose, the largest normed eigenvalues are determined, and associated eigenvectors are used to project the dataset into a lower-dimensional space. The ideal dimension of the projected dataset is a crucial point. In this work, we considered the decay of the eigenvalues to determine the optimal dimension for the PCA. Besides that, by considering the small sample number, projections into very low-dimensional spaces were also examined.

##### Wavelet Transform

The theory of wavelets emerged in the 1990s to address some limitations of classical Fourier analysis, namely, its limitations in providing local features of signals. The advantage of the wavelet transform is its ability to capture the local properties of the signals, on the contrary to Fourier transform. Consequently, in the last decade, wavelet transform is mostly preferred for analysis when the local information matters. Besides, it is shown that the wavelet transform is very successful in finding the most informative representation of the data in the classification tasks (al-Qerem et al., 2020). Consequently, in many studies, wavelet coefficients are used both for dimension reduction and feature selection purposes (Hazarika et al., 1997; Qu et al., 2003).

Several properties of the wavelet system, including regularity and fast decay, can be controlled by the choice of mother function (Wojtaszczyk, 1997; Heil, 2010). For the given study, we preferred to use Daubechies as the mother function of order four. These parameters are determined numerically to have maximal efficiency during the computational analysis. After the wavelet coefficients were calculated, the 10% largest coefficients were collected to represent the samples. The optimal number of coefficients to be used was again determined by optimizing the efficiency of the numerical analysis.

##### Stepwise Linear Discriminant Analysis

Linear discriminant analysis (LDA) is one of the basic dimension reduction and supervised data classification techniques. The idea of LDA is finding a separating hyperplane by maximizing the inter-class variance and minimizing the intra-class variance (Bishop and Nasrabadi, 2006). Through that idea, LDA has the capability to form a successful separating hyperplane even in the cases, PCA fails. Hence, LDA has several applications in data analysis fields both for dimension reduction and for data classification. Stepwise linear discriminant analysis (SWLDA) forms this separating LDA hyperplane step-by-step to find the set of features to form the best separating hyperplane. Hence, SWLDA is a linear classifier and determines the most informative features at once.

In our analysis, we applied SWLDA both as a feature selection technique and as a linear classifier. For that purpose, we used the toolbox by brain–computer interface (BBCI) (Blankertz et al., 2016). Due to the mathematical theory of LDA, we cannot select more features than the number of samples. Hence, the maximum number of features to be selected was set to the number of training samples.

##### Support Vector Machine Classifier Weights

Support vector machine (SVM) is a model-based supervised data classification technique. The main idea of SVM is to specify a linear subspace that separates samples of two different classes with a maximum margin. It generates a decision function and clusters each subject to one side of the determined subspace. The source of the high success of SVM lies in its ability to handle high-dimensional datasets and the logic called kernel trick. If the data cannot be linearly separated in its own space, it is transferred to another space with a kernel function. This space is where the data can be separated from each other in the most optimal way, theoretically, this space does not need to be known by the user, and mathematical operations can be done without any knowledge of the space (Burges, 1998). SVM outputs the decision function that has the form:


$$\begin{array}{l} f\left(x\right)=\overset{N}{\underset{m=1}{\sum }}a_{m}y_{m}K\left(x,x_{m}\right)+b\; \end{array}$$

Here, N is the number of support vectors, α_m_ is the weighted components, y_m_ is the label of the sample x_m_, b is the tolerance constant special for each class, and K is the kernel function. The K kernel function is a form of similarity measurement that plays a key role in representing and decomposing the data in space and is defined as follows:


$$\begin{array}{l} K\left(x_{i},\;x_{j}\right)=\;<\varphi \left(x_{i}\right),\;\varphi \left(x_{j}\right)> \end{array}$$

In addition, considering the decision function equation, it is seen that the α_m_-weighted components with the kernel function, K(x,x_m_), directly affect the decision. Hence, the absolute value of the feature weights assigned by the SVM model is an indicator of the importance of features on the decision. In our study, this idea played a critical role in investigating which features were important in the decision mechanism. By maximizing the classification accuracy, the features were arranged in the order of importance.

Since we focused on the multi-source nature of the EEG signal, we carefully applied all feature extraction transformations separately for each source of EEG data. By that strategy, we avoided any miscalculation due to the discontinuity between subsequent sources.

#### Data Classification

For the classification task, we focused on SVM classifier due to its multiple advantages over other state-of-the-art classification techniques. Widely used machine-learning techniques in EEG analysis, which are random forest classifier, naïve Bayes classifier, and decision tree, have limited capability to handle such a complicated task (Wang et al., 2019; Anuragi and Sisodia, 2020; Roy et al., 2021). These methods have a low tolerance to high variance and high-dimensional data. More sophisticated techniques such as artificial neural networks (ANN) have more capability to handle such challenging tasks (Roy et al., 2019). However, these methods tend to overfit when the sample number is limited. Once a large dataset is available, ANN would work great, but it is so difficult to avoid overfitting ANN models for such an asymmetric dataset as EEG. However, SVM has the capability to handle difficult tasks such as high variance and high dimensionality (Bishop and Nasrabadi, 2006). More importantly, SVM allows us to extract the feature hierarchy from the model. Hence, we have access to the information on how significant each feature is for the decision-making process. With all these advantages, we decided that the SVM classifier is the best option for the aimed task. In SVM analysis, we used radial basis function (RBF) kernel, and the sigma value was determined numerically for each case. On the other hand, to validate our strategy for data classification, we compared the performance of SVM with two ANN architectures EEGNet (Lawhern et al., 2018) and Shallow ConvNet (Schirrmeister et al., 2017), which are specialized for EEG analysis, and a more traditional approach SWLDA classifier. All parameters such as filter size, stride, and pooling size were optimized according to the sampling rate by the guide of references. Dropout value was optimized for each run to the value of 25 or 50%. Learning rate was optimized for each run. Each model is trained for 150 epochs.

The total number of 14 patients in our dataset was distributed unevenly into two groups. The number of TMS+ patients was 8 whereas the number of TMS– patients was 6. Due to the limited sample number and the risk of large biological variance, observing the unbiased results was crucial. For that purpose, we always used the Monte Carlo technique during the analysis. The Monte Carlo technique basically estimates the numerical results of non-deterministic events by relying on multiple randomly selected sample spaces (Bauer, 1958). It bases on the randomness to observe the actual routine of the events, which is a core concept in probability theory. It is widely used for data analysis especially for biological data science when the sample number is limited (Manly, 2018). Here, the main goal is covering all possible outcomes through the random trials. Hence, using the combination theory, we calculated the optimum number of random trials that needed to be performed to collect information from all possible sampling. All reported results are the average of the success rates of the optimum number of trials with random data selections. All random sets are selected by the normal distribution, so we can assume that all possible combinations of the samples were considered. As a result, given numerical values are the best estimation of the actual performance of the classification model. Furthermore, this technique based on randomness enables us to calculate the variation in the dataset.

For further analysis, we focused on the stability and the sensitivity reasoning of this study with respect to sample size and uneven distribution of samples to different groups. The sensitivity and stability analyses were done on EEG2 with SVM classifier. With the purpose of measuring the effect of unevenly distributed sets, we compared the classification results of balanced and unbalanced training sets. An unbalanced validation data were causing results to fluctuate wildly, hence harming the model optimization process. Similarly, with an unbalanced test data, numerical results were misleading about the quality of the model. Hence, the numerical analysis was performed always with balanced test and validation datasets.

The size of training and validation sets are the important aspects. By optimizing these values, the required sample size would be determined, and the computational studies can be optimized for the targeted tasks. To see the effect of the size of validation and training datasets, we repeated classification analysis with different dataset sizes and reported the results in the Results section.

During the classification analysis, we applied for the leave-one-out cross-validation since the dataset size was limited. For that purpose, we chose one patient as a test sample, and the remaining samples were used to form a balanced train and validation sets (Hastie et al., 2009). From the remaining 13 patients, over 30 random selections, we selected a maximal balanced set. The balanced dataset was divided into two sets such as training and validation sets.

After observing satisfying results by the leave-one-out validation, we continued our analysis with direct classification despite the small dataset size. However, during the data classification process, due to a small sample number, we could not use a validation data. We split the dataset into training and test sets. This model may not be generalizable due to the limited sample size, but the results can be considered as an indication of the feasibility of the task.

##### Optimization of SVM Parameters

The selection of the SVM parameters is very critical for the performance of the method. SVM works on high-dimensional spaces and may tend to overfit training data. In the literature, the grid search is the most widely used technique for optimizing SVM parameters. The grid search is based on generating a grid by all possible combinations of parameters. In the generated grid, all values are examined and the combination which yields the maximum classification result is determined as the optimum set of parameters. Since all possible parameters are checked, theoretically, the most optimum results will be determined by the grid search. However, for our case, since we applied Monte Carlo technique, we had to perform parameter optimization several times which is computationally expensive and time-consuming. Hence, in our study, we used an evolutionary algorithm to optimize the parameters (Syarif et al., 2016). Our parameter optimization algorithm is an iterative method, starting with user-determined step size and minimum and maximum values for each parameter. In every iteration, it updates the search interval accordingly and reduces the step size to find the optimum parameters. Once the accuracy values start decreasing, it is considered as a stopping criterion. In contrast to grid search, this method does not check all possible combinations. However, the computational complexity is significantly reduced. In the literature, such genetic algorithms were shown to estimate the optimum values by grid search with a minimal error (Syarif et al., 2016). This process was repeated for each random trial, so for each run in Monte Carlo, we used the optimal parameters for the SVM classifier.

## Results

**Figure 4:**
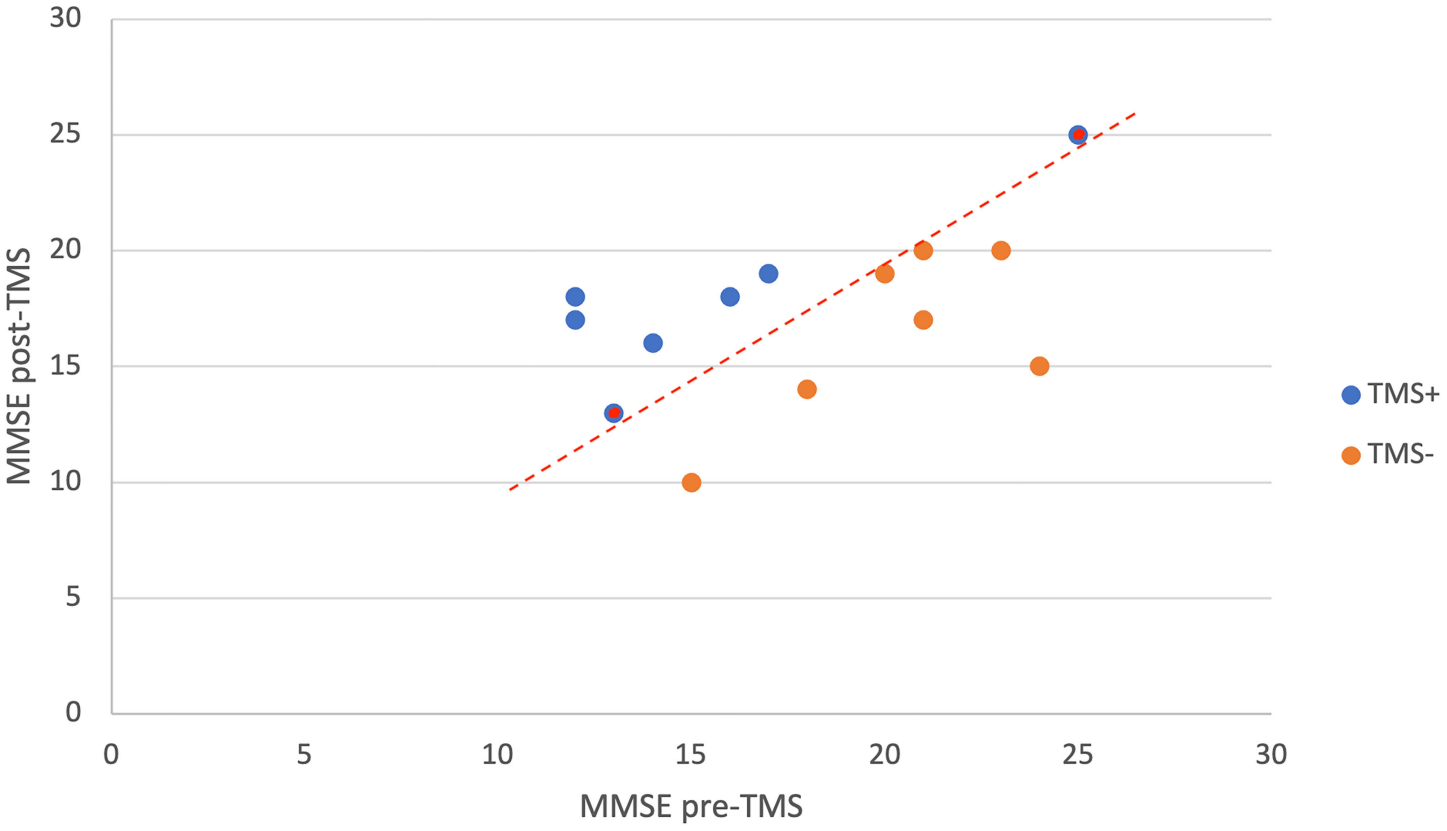
The change in MMSE scores of TMS+ and TMS- patients with the TMS application. As it is seen in the figure, these two groups can be separated from each other with the red dashed decision line. The patients with the same MMSE score post- and pre-TMS which are labeled as TMS+ are represented as red dots inside blue.

All patients had mild to moderate AD symptoms (CDR = 1–2). The mean cognitive scores and demographic features of the patients are shown in Table 2. The change in MMSE scores before and after the TMS application in TMS+ and TMS– patients can be seen in Figure 4. Between two groups (MMSE pre-TMS and MMSE post-TMS), one-way ANOVA was applied, and statistically significant results were observed in MMSE pre-TMS group (*F* = 5.153, *p* < 0.05). According to *post hoc* analysis, statistical difference has been obtained between TMS+ and TMS– (*t* = −2.27, *p* < 0.05).

**Table 2:** Baseline demographic characteristics of the patients.

N	14
Age	69.86 ± 8.23
Sex (male/female)	5/9
MMSE score (pretreatment)	18.09 ± 4.74

**Figure 5:**
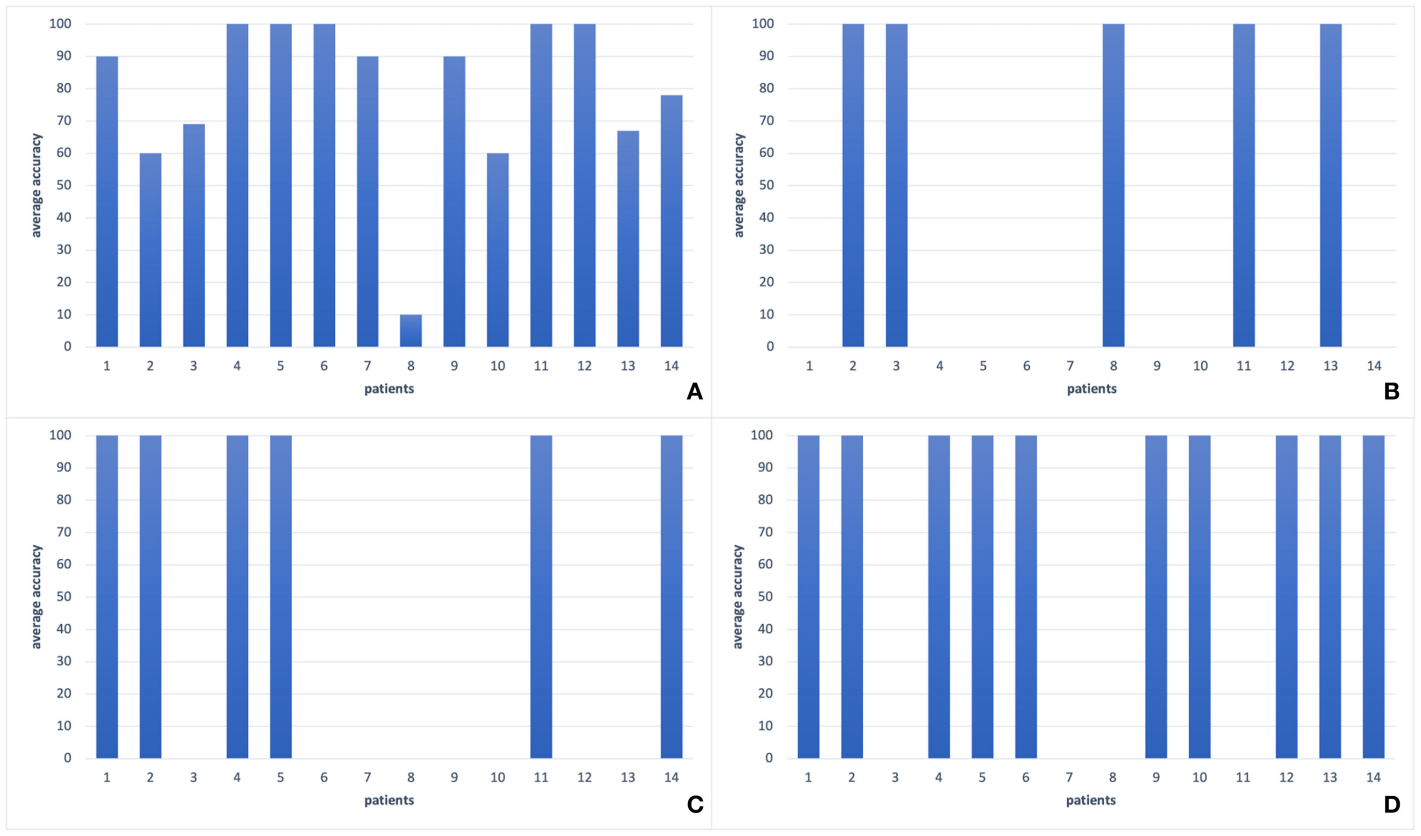
The comparison of different classification methods with leave-one-out validation of EEG2 signals of each patient in our dataset. **(A)** SVM classifier (78%), **(B)** SWLDA classifier (36%), **(C)** EEGNet (43%), **(D)** Shallow ConvNet (71%). As it is seen, there is a large variance among patients for all classification methods. However, most of the patients form a coherent team whereas two patients' accuracies are around 60%, and one has an accuracy about 10%. The best two performances can be seen by SVM and Shallow ConvNet.

For the feature selection process, we applied PCA, wavelet transform, and SWLDA, and by the lead of SVM and SWLDA weights, we used only theta band features. We compared the classification accuracies with these different feature selection techniques (Table 3) where the classification accuracy is calculated as the percentage of the number of correctly classified samples to the total number of samples. For the classification analysis, we applied SVM, EEGNet, Shallow ConvNet, and SWLDA and compared their performances (refer to Table 4; Figure 5).

**Table 3:** Comparison of performance of feature selection methods with SVM leave-one-out validation of EEG2.

	**No feature selection method**	**PCA**	**Wavelet transform**	**SWLDA**	**Theta band features**
EEG2	78%	62%	67%	58%	**93**%

*As it is seen, whole theta band features give significantly higher accuracy than other methods. The maximum accuracy is in bold*.

**Table 4:** Comparison of performance of classification methods with power spectra features of EEG2.

	**SVM**	**ShallowNet**	**EEGNet**	**SWLDA**
Leave-one-out validation	**78**%	**71**%	43%	36%

*The maximum accuracies for each category are in bold*.

**Figure 6:**
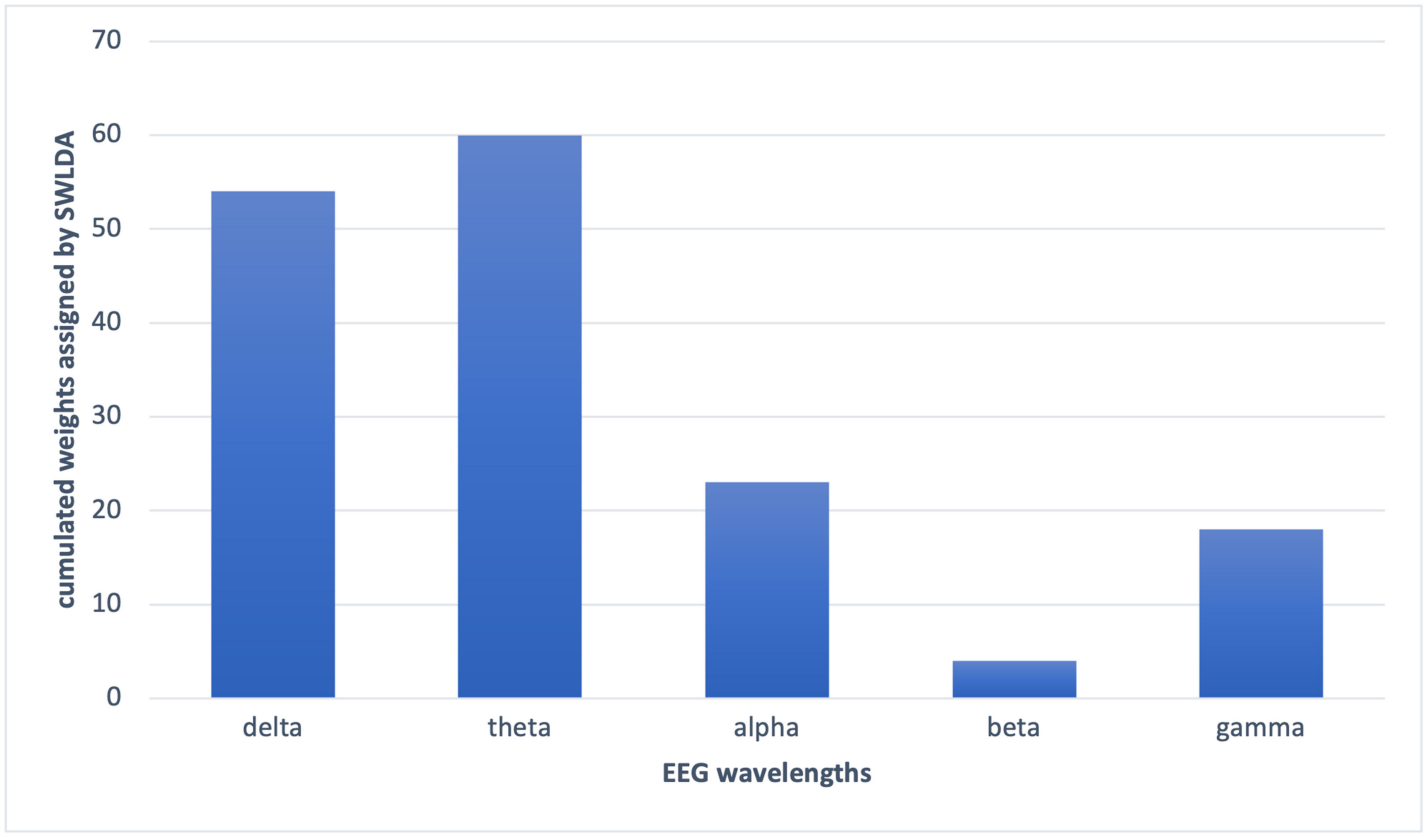
The cumulated weights assigned by SWLDA to each wavelength features. SWLDA mostly selected theta band features as informative and assigned the largest weights to the theta band features.

In the PCA, the decay of eigenvalues showed us that most of the information was stored in 20 features only. Using the most informative 20 features selected by PCA, we got an average of 62% accuracy on leave-one-out validation of EEG2. In the wavelet transform application for the feature selection purpose, by optimizing the numerical analysis, we decided to keep the largest 10% of the wavelet coefficients. Through that, we got 67% on the leave-one-out validation of EEG2. In the SVM and SWLDA, we observed that the average weights of theta wavelength were remarkably higher than the others (Figure 6). We used this idea as a lead and measured the performance of the data classification task using features collected only from theta wavelength which yield the best classification performance. In EEG1 analysis, we observed that none of the wavelengths nor the locations were given significantly more information than others.

To measure the effect of using the multi-source form of EEG signals, we analyzed the dataset with the power of each wavelength. As a result, we got 42% of accuracy overall for leave-one-out validation of EEG2. Hence, keeping the signals collected from each location gives significant information for classification purposes.

We observed the highest classification performance as 93% with SVM on theta band features. As it is seen in Table 5, regardless of the classification or feature selection technique, we always got better performance with EEG2 than EEG1. The average performance of the leave-one-out validation of EEG2, without any further feature selection or dimension reduction method, was recorded as 78%. In this analysis, we have seen that most of the patients always give a classification accuracy >50%. Always, the same patients had poor classification accuracies. For example, one of the patient's average EEG2 classification rate was about 10% (Figure 5A), which means that this patient information does not fit on the general of the other patients. However, we observed that the majority of the patients form a coherent team and give more than 90% of accuracy each. On the other hand, in the EEG1 analysis, we recorded 68% as the average accuracy and the coherence between the samples was lower. Furthermore, the gain by the feature selection and dimension reduction methods was higher in EEG2 analysis. Through the feature selection method application, the average performance of the leave-one-out validation of EEG2 increased to 93%, while we obtained 79% of accuracy on validation of EEG1.

**Table 5:** All given accuracies are the result of the Monte Carlo Technique.

**Feature vector**	**Power spectra features**		**Theta band features**	
**Validation technique**	**Leave-one-out**	**Direct classification**	**Leave-one-out**	**Direct classification**
EEG1	68%	**64**%	**79**%	63%
EEG2	78%	77%	**93**%	**82. 5**%

*In the table, it is seen that EEG2 gives better results than EEG1 for all cases. Feature selection technique also improves the results. The maximum accuracies for each category are in bold*.

After observing satisfactory results in the leave-one-out validation, we continued with the classification model generation. We obtained 82.5% of accuracy on the EEG2 test data as an average by the Monte Carlo technique, and 64% of accuracy on the classification of EEG1, by the same procedure. During our classification model generation, we observed that the optimum training sample ratio was about 65% (9 samples out of 14). As the mean of 50 random trials, we got 82.5% of accuracy while the standard deviation was 12.5%. So, the minimum accuracy was about 75%, which means that we got 3 correct predictions out of 4 on average by EEG2 analysis.

## Discussion

As a conclusion of our analysis, we observed that it is possible to predict the response of patients with AD to the TMS treatment by both EEG1 and EEG2 analyses. However, EEG2 is more informative than EEG1 with an average accuracy of 93 % while we obtained 79% on EEG1. Besides, due to our limited sample number, we focused on the data analysis and investigated the challenges of the EEG data analysis on patients with AD. We measured the feasibility of generating a personal TMS treatment route for the patients with AD and showed the possibility is higher if we use the lead of EEG signals after rTMS application. In addition to that, this type of algorithm, which can predict which patient will benefit from the TMS application by using the EEG data, will not only prevent patients from losing time with TMS treatment, but also will save on health expenditures. Thus, it will be possible to use TMS more effectively in neurodegenerative diseases such as Alzheimer's disease.

Due to its non-invasive nature, relative ease of administration, and low cost compared to other neuroimaging methods, EEG represents a promising tool for developing a personalized medicine approach for rTMS treatment (Garnaat et al., 2019). However, there is still a very limited number of studies in this area.

One of the previous studies that tried to distinguish responders from non-responders to rTMS treatment in depression using resting-state EEG analysis differentiated these groups and retrospectively identified several pretreatment variables such as higher anterior individual alpha frequency values, lower power in the frontocentral theta frequency band, and increased prefrontal delta and beta values (Arns et al., 2012). However, a repeat study to confirm these promising results found no significant differences between responders and non-responders in terms of individual alpha frequency, frontal theta, even after controlling for sex or age (Krepel et al., 2018; Garnaat et al., 2019).

There are only a few studies on machine-learning analysis of EEG in predicting the effectiveness of rTMS. All these attempts focus on depressive disorders. Although sophisticated methods are introduced, since the TMS therapy for patients with AD have more factors to be considered than depressive disorders, the given methods are weak to handle the current task.

In prospective studies, there were only a few possible predictors of the outcome for rTMS treatment for depression patients, and further research will be required to translate any of the findings described above into guidance for the implementation of treatment (Garnaat et al., 2019).

Our findings are particularly parallel to the findings by Bailey et al., He also reported that alpha and theta bands are useful to predict benefiting from rTMS therapy for depression (Bailey et al., 2019). However, in another study, they conducted with a larger and different sample group, and they could not replicate these findings for alpha and theta (Bailey et al., 2021). As it is known, the main element of the changes in EEG in Alzheimer's disease is the changes in alpha and theta (Bhattacharya et al., 2011; Güntekin et al., 2020). The results of our study are compatible with this general framework. There are also some findings showing that EEG event-related theta responses may be sensitive to neuromodulation interventions (Cespon et al., 2019; Güntekin et al., 2020). However, the impaired dynamics associated with EEG in AD are not unique only to theta and alpha. Again, it is clear that the localization of rTMS applications can cause responses at different frequencies by many different mechanisms. Indeed, Koch et al., showed that rTMS intervention on the precuneus for 2 weeks modulated only beta responses in AD (Koch et al., 2018). For this reason, there is a need for larger sample groups and further research on patients with other frequencies.

### Justification of Sample Size for the Data Analysis

For the aimed data analysis task, considering the small sample number with the large feature vectors, getting a unique solution was not possible. Instead, we got statistical confidence by applying the Monte Carlo technique. For that purpose, we repeated the analysis with multiple randomly selected datasets with varying sizes and combinations of samples. Through that, we obtained the best estimation of the actual performance of our model despite the limited sample size. Still, to have theoretically justified results, we repeated the analysis with the low number of features. For that purpose, we applied different dimension reduction techniques. However, we observed that a low feature number does not satisfy a qualified data classification model for the current dataset.

We observed that SVM works better than EEGNet, Shallow ConvNet, and SWLDA for the classification of our dataset. Since the task is challenging, the low feature number would not store the required information. As a result, SWLDA failed in the analysis. The ANN architectures such as Shallow ConvNet and EEGNet tend to overfit for our dataset with a small sample number. However, Shallow ConvNet showed a close performance to SVM classifier. This would lead that with a larger dataset, a shallow convolutional neural network would give promising classification results.

**Figure 7:**
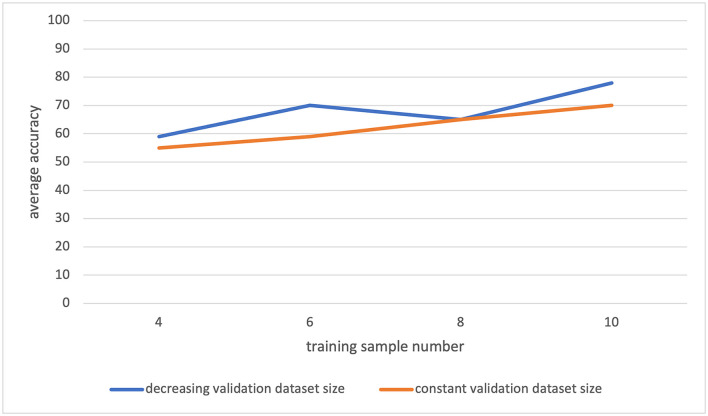
The leave-one-out classification result for varying training and validation dataset sizes. The orange plot: for different training dataset sizes, the validation dataset is always formed by two samples. The blue plot: for different training dataset sizes, the validation dataset is formed as the remaining samples after the test and training sets are generated.

During the data analysis, we measured that the intra-class variance is large. As a result, the selection of the training samples was significantly changing the classification accuracy. We have seen that in a class (TMS+ or TMS–), some patients' information conflicts with each other. So, the inclusion of one of these patients in the training dataset was leading to a model which would mislabel the other patient. This information supports the large variance in the dataset. Besides, as it is seen in Figure 7, with increasing training dataset size, the classification accuracy increases and has not stabilized with the maximal training set size. This suggests that we have not reached the required sample number yet. Hence, the data analysis indicates that more patients are required to generate a more generalizable model.

**Figure 8:**
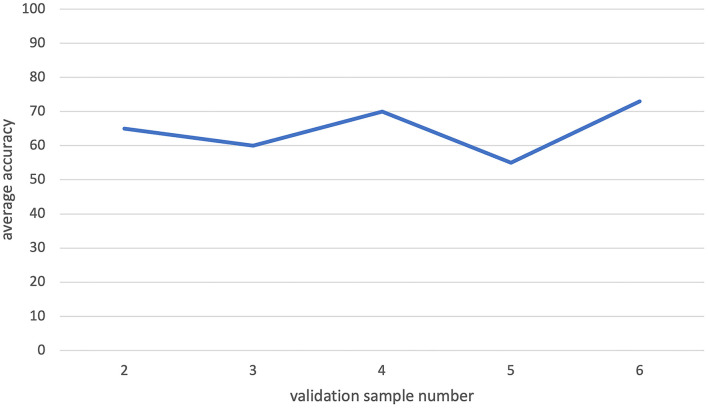
The leave-one-out classification result for increasing validation dataset sizes while training size is fixed. There is no visible increasing pattern in the graph. So, increasing validation size does not have a significant effect on the classification process.

We observed that the increasing validation data size may have a negative effect on the classification performance in some cases (Figure 8). The reason for that may be the fact that some patients' information totally conflicts with others, and hence, the inclusion of certain patients in the validation data would cause a decrease in classification accuracy. When we use smaller validation data, the effect of these certain patients would decrease. Hence, smaller validation data with a large training dataset gave the largest accuracy during our analysis.

By considering the classification analysis results (Table 5), we concluded that the EEG2 signals are more informative than the EEG1 to recognize TMS+ and TMS- patients. Similarly, EEG2 has more capacity to be improved by feature selection processes. Hence, EEG signal collected after a TMS therapy would lead the more efficient prediction models for the personalized TMS treatments for patients with AD.

For the future direction, this analysis must be repeated with a larger dataset. This will increase the validity of the data analysis results. We also observed that common data augmentation techniques for signals did not supply any improvement in the classification performance. In the literature, it is seen that for different neurological tasks, different types of data augmentation techniques must be applied (Lashgari et al., 2020). Hence, for future studies, the best augmentation techniques for the Alzheimer's disease must be determined, and those techniques must be applied to the present data. Through that, a more reliable and consistent classification model could be presented. Similarly, using a multi-kernel technique such as MKSVM instead would produce a better result due to the multi-source nature of our dataset. Besides that, a shallow artificial neural network would increase efficiency with its capability to handle challenging tasks.

In this study, due to the limited sample number and observed biological variance, we mostly focused on the investigation of the data from various perspectives. We measured the feasibility of generating a personal treatment route for the TMS treatments for patients with AD and showed that the possibility is high. However, for getting a reliable and less risky decision model, we need to have a better observation of the biological variance, which requires more data.

## Data Availability Statement

The raw data supporting the conclusions of this article will be made available by the authors, without undue reservation.

## Ethics Statement

The studies involving human participants were reviewed and approved by the Ethical Committee of Istanbul Medipol University the number of Ethical Report: 10840098–604.01.01–E.45425. The patients/participants provided their written informed consent to participate in this study.

## Author Contributions

LH designed and supervised all the experiments. HV conceived the experiments. CK designed the figures and analyzed and performed the machine-learning analysis. All authors performed the experiments and/or analyzed the data. All authors contributed to the interpretation of the results, writing the article, and approved the submitted version.

## Funding

Istanbul Medipol University support for researchers for submission expenses.

## Conflict of Interest

The authors declare that the research was conducted in the absence of any commercial or financial relationships that could be construed as a potential conflict of interest.

## Publisher's Note

All claims expressed in this article are solely those of the authors and do not necessarily represent those of their affiliated organizations, or those of the publisher, the editors and the reviewers. Any product that may be evaluated in this article, or claim that may be made by its manufacturer, is not guaranteed or endorsed by the publisher.
